# The role of interferon-stimulated long non-coding RNA PKR-IT1 in the regulation of Zika virus and dengue virus replication and antiviral response

**DOI:** 10.1128/spectrum.01148-26

**Published:** 2026-09-24

**Authors:** Yi Wang, Ai Fu, Liping Chen, Yue Shi, Qian Chen, Fei Wang, Qian Huang, Shourong Liu

**Affiliations:** 1 Clinical Research Laboratory, Affiliated Hangzhou Xixi Hospital, Zhejiang Chinese Medical University 70571, Hangzhou, China; 2 Department of Infection, Affiliated Hangzhou Xixi Hospital, Zhejiang Chinese Medical University 70571, Hangzhou, China; Thomas Jefferson University, Philadelphia, Pennsylvania, USA

**Keywords:** flavivirus, interferon, lncRNA, viral replication, innate immunity

## Abstract

Interferon-stimulated genes play a critical role in the innate immune response against flaviviral infection. While recent studies have reported that interferon-stimulated lncRNAs participate in antiviral immunity, the functions of numerous lncRNAs in host-flavivirus interactions remain uncharacterized. Here, we demonstrate that the expression of lncRNA PKR-IT1 is induced by ZIKV and DENV infection. PKR-IT1 was induced by virus, poly(I:C), and IFNs in A549 cells, but this induction was largely abolished upon knockout of IPS-1 or IFNAR1 during viral infection. Additionally, PKR-IT1 overexpression inhibited viral production, whereas its suppression in A549, Huh7, and SH-SY5Y cells enhanced ZIKV replication. In PKR-IT1 knockout cells, viral replication also markedly increased compared with that in control cells. Interestingly, PKR-IT1 overexpression enhanced the activation of the JAK-STAT signaling pathway triggered by flaviviral infection and upregulated ISG expression. Conversely, PKR-IT1 deficiency decreased JAK1 and STAT1 phosphorylation levels, thereby impairing ISG expression. Mechanistically, the silencing of STAT1 in PKR-IT1-overexpressing cells partially restored ZIKV replication. Furthermore, PKR-IT1 directly bound PABPC1 and promoted its proteasome-dependent degradation. PABPC1 was found to bind to ZIKV RNA, while the inhibition of PABPC1 attenuated the levels of ZIKV protein, viral particles, and later viral RNA. Notably, the reintroduction of PABPC1 restored viral replication in PKR-IT1-overexpressing cells. Clinically, PKR-IT1 levels negatively correlate with fever duration and symptom number, and are lower in DENV-infected patients with severe leukopenia and thrombocytopenia. In summary, PKR-IT1 modulates flavivirus replication and JAK-STAT signaling, and may serve as a clinical correlate of dengue severity, offering insights for antiviral therapy development.

**IMPORTANCE:**

Flaviviruses such as ZIKV and DENV pose significant public health threats, yet the roles of long non-coding RNAs (lncRNAs) in host antiviral immunity remain poorly understood. Here, we identified that PKR-IT1, as an interferon-stimulated lncRNA, not only suppresses flavivirus replication by binding PABPC1 to promote its proteasome-mediated degradation, but also enhances the activation of the JAK-STAT signaling cascade during flavivirus infection. Importantly, clinical correlations reveal that lower PKR-IT1 expression is associated with severe leukopenia and thrombocytopenia in DENV-infected patients, as well as longer fever duration and more symptoms. These findings establish that PKR-IT1 not only acts as a novel modulator of both flavivirus replication and the JAK-STAT signaling pathway, but also may serve as a correlate of dengue disease severity. By elucidating a previously uncharacterized lncRNA-mediated regulatory mechanism, this work provides a foundation for developing therapeutic strategies and prognostic tools against flaviviral infections.

## INTRODUCTION

Flaviviruses are single-stranded, positive-sense RNA viruses, comprising, among others, Zika virus (ZIKV), dengue virus (DENV), and Japanese encephalitis virus (JEV). Primarily transmitted to humans through mosquito vectors, these viruses infect over 400 million people globally each year and pose a serious threat to public health (1). Effective and broad-spectrum therapeutics against flaviviruses are currently lacking, highlighting the urgent need to identify novel antiviral targets. Host factors involved in the regulation of viral replication represent promising targets for antiviral drug development. Given that a single host factor is capable of modulating the replication of multiple flaviviruses, targeting these factors offers a potential avenue for designing broad-spectrum and potent antiviral therapeutics (2).

Studies have demonstrated that interferon (IFN)-stimulated protein-coding genes, such as poly(ADP-ribose) polymerase family member 12 (PARP12) (3), NLR family pyrin domain containing 12 (NLRP12) (4), protein kinase regulated by dsRNA (PKR) (5), tripartite motif containing 69 (TRIM69) (6), TRIM22 (7), and ISG15 (8), can significantly inhibit the replication of various flaviviruses. Interferon-stimulated genes (ISGs) also comprise IFN-stimulated long non-coding RNAs (lncRNAs; ISRs) (9). LncRNAs are functional RNA molecules longer than 200 nucleotides that lack protein-coding capacity. They typically adopt complex secondary structures, are expressed at low abundance, and display marked species- and tissue-specific expression patterns (10). Functionally, lncRNAs regulate host antiviral innate immune responses at transcriptional, translational, and chromatin levels (9), and are thus considered a promising area of research for deciphering the mechanisms underlying virus-host immune interactions.

Recent studies have demonstrated that ISRs play significant roles in the innate immune defense against RNA viruses, including flaviviruses. ISRs can exert positive or negative feedback on IFN signaling pathways through diverse mechanisms, thereby indirectly modulating RNA virus replication (9). The functional mechanisms via which ISRs modulate antiviral innate immunity primarily fall into three categories: regulation of pattern recognition receptor activation (11–13), modulation of IFN production (14–18), and control of ISG expression (19–26). Whether ISR-mediated regulation of the innate immune response to RNA viruses involves additional molecular mechanisms merits further investigation.

Studies have demonstrated that lncRNAs activate the JAK-STAT signaling pathway, resulting in specific biological outcomes (27). Currently described activation mechanisms primarily involve: (i) lncRNAs binding to RNA-binding proteins (RBPs), thereby enhancing the stability of mRNAs encoding key JAK-STAT signaling molecules, such as STAT1, and leading to pathway activation (28); (ii) lncRNAs competitively binding to and inhibiting the expression or function of negative regulators of the JAK-STAT pathway, such as suppressor of cytokine signaling 1 (SOCS1), resulting in sustained pathway activation (29); and (iii) lncRNAs functioning as competing endogenous RNAs (ceRNAs) that sequester microRNAs (miRNAs), thereby upregulating the expression of key JAK-STAT components such as STAT1 and enhancing pathway activity (30).

The lncRNAs currently known to activate the JAK-STAT pathway are not classified as ISRs within the existing literature. It is well established that in IFN-mediated innate immunity, IFNs activate the JAK-STAT signaling pathway following receptor binding, leading to the induction of ISG transcription. However, whether ISRs can also regulate the activation of the JAK-STAT pathway induced by RNA viruses remains unknown.

Previous studies verified that the function of lncRNA is generally associated with its co-expressed adjacent protein-coding gene (31). In this study, based on previous lncRNA microarray data (14), a novel host intronic lncRNA co-expressed with the adjacent antiviral ISG PKR (32) was identified. We identified a PKR intronic RNA 1, designated PKR-IT1, and showed that its expression was upregulated upon ZIKV infection. Further investigation demonstrated that PKR-IT1 is a novel ISR, and its induction is dependent on the innate immune response. Importantly, PKR-IT1 both potentiates JAK-STAT signaling to induce ISG expression and promotes PABPC1 degradation via proteasome, thereby suppressing flavivirus replication. Extending our findings to the clinical setting, we observed that PKR-IT1 levels were significantly elevated in DENV-infected patients compared with healthy controls. Our further analysis revealed that PKR-IT1 levels were significantly negatively correlated with fever duration and the number of symptoms; moreover, PKR-IT1 expression was significantly lower in DENV-infected patients with severe leukopenia and thrombocytopenia. These results not only provide novel insights into the role of PKR-IT1 in regulating flavivirus replication and JAK-STAT signaling activation, but also represent a potential tool for the clinical diagnosis of severe leukopenia and thrombocytopenia.

## MATERIALS AND METHODS

### Cell culture

The A549, Vero, BHK21, 293T, Huh7, HepG2, and SH-SY5Y cell lines used in this study (ATCC) were maintained in Dulbecco’s modified Eagle’s medium (Gibco) supplemented with 10% fetal bovine serum (Gibco), penicillin (100 U/mL), and streptomycin (100 µg/mL) at 37°C under a humidified atmosphere with 5% CO_2_. IPS-1 and IFNAR1 knockout cell lines were generated using the clustered regularly interspaced short palindromic repeats (CRISPR)/CRISPR-associated protein 9 (CRISPR-Cas9) system as previously described (33, 34).

### Viruses, viral infection, and viral titration

The ZIKV (H/PF/2013 strain), DENV2 New Guinea C, and DENV16681 were kindly provided by Prof. Ping Zhang (Zhongshan School of Medicine, Sun Yat-Sen University). Viruses were propagated in Vero cells, and virus-containing supernatants were collected following centrifugation. Virus stocks were titered on Vero cells (ZIKV) or BHK21 cells (DENV2 and DENV16681) using a previously described (14, 35, 36) standard plaque assay and stored at −80°C. Cells were infected with ZIKV, DENV2, or DENV16681 at an MOI of 1. For the single-step virus growth assay, the supernatants were harvested at 24 h post-infection and subjected to viral titration using the standard plaque assay.

### Functional annotation of PKR-IT1 target mRNAs

DAVID Bioinformatics Resources (version 6.7, https://davidbioinformatics.nih.gov/) was used to perform functional annotation enrichment as well as to assess the enrichment of input genes through gene ontology (GO) term and Kyoto Encyclopedia of Genes and Genomes (KEGG) pathway analysis.

### Quantitative real-time PCR (qPCR)

Total RNA was extracted using TRIzol reagent (Invitrogen) and reverse-transcribed into cDNA using HiScript II Reverse Transcriptase (Vazyme). qPCR was performed with SYBR Green Master Mix in a Bio-Rad FQD-96A cycler, and gene expression levels were normalized to ACTIN. The sequences of the primers used for qPCR are listed in Table S1. Differences in gene expression were calculated based on ΔCT values as previously described (14).

### dsRNA and IFN treatment

A549 cells were transfected with 0.4 μg/mL poly(I:C) (MCE) using Lipofectamine 2000 (Invitrogen) for 12 h or treated with 100 U/mL IFN-β (MCE) for 20 h.

### Plasmid construction

The single-guide RNAs (sgRNAs) targeting PKR-IT1 were inserted into the lentiCRISPR v2 vector (Addgene, #52961). PKR-IT1 PCR fragments were cloned into the pcDNA3.1(+) and pSG5 vector, yielding pcDNA3.1(+)-PKR-IT1 and pSG5-PKR-IT1-FLAG. All primer and sgRNA sequences are listed in Table S1. PABPC1 expression plasmids were obtained from MIAOLING BIOLOGY (China, Hubei). The sequences of all plasmids were verified by sequencing.

### Generation of PKR-IT1-expressing cells

A549 cells were transfected with pcDNA3.1(+) vector or pcDNA3.1(+)-PKR-IT1 using Lipofectamine 2000 (Invitrogen). Control cells and PKR-IT1-expressing cells were selected with G418 for 7–10 days. The efficiency of gene overexpression in cells was measured by qRT-PCR.

### Generation of PKR-IT1 knockout cells

293T cells were transfected with lentiCRISPR carrying PKR-IT1 sgRNA together with pSPAX2 and pMD2.G using FuGENE HD Transfection Reagent (Promega). Supernatants were collected at 2 days post-transfection and were employed for A549 cell transduction. Knockout cells were selected using puromycin and limiting dilution cloning. To validate the knockout effect, genomic DNA of cloned cells was extracted using a cell genomic DNA extraction kit (Biotek). Regions surrounding sgRNA target sequences were amplified by PCR and cloned into pMD-18T (Takara) for sequencing. The efficiency of gene knockout in cells was measured by qRT-PCR.

### RNA interference

Small interfering RNAs (siRNAs) were sourced from Gene Pharma (Shanghai, China). The sequences of the two siRNAs targeting human PKR-IT1 RNA were GAUCGUUAAUUUACACAGA and GCAAGACUAUGGAAAGGAA. The sequences of the two siRNAs targeting human PABPC1 RNA were GUAGGCAACAUAUUCAUUA and CACCUCACUAACCAGUAUA. A control siRNA with scrambled sequence was used as a negative control (siNC). Transfection was carried out with 2 μmol siRNAs using Lipofectamine 2000 reagent (Invitrogen) according to the manufacturer’s instructions.

### Proteasome inhibitor treatment

MG132 was purchased from MedChemExpress Biotechnology (CAS number: 133407-82-6). Stock solutions of MG132 were prepared in DMSO at a concentration of 10 mg/mL. A549 cells were transiently transfected with the pcDNA3.1(+) vector and pcDNA3.1(+)-PKR-IT1 for 24 h and treated with MG132 (25 mM) for an additional 12 h; the groups of control cells treated with DMSO were evaluated in parallel with the experimental groups. Then cell lysates were subjected to western blot, as previously described (37).

### Western blot

Whole-cell extracts were prepared in RIPA lysis buffer. Proteins were separated by 10% SDS-PAGE (Epizyme Biotech) and then transferred onto PVDF membranes (Millipore). After blocking with 5% fat-free milk in PBST at 25°C for 2 h, the membranes were incubated overnight at 4°C with primary antibodies, including anti-ZIKV envelope (BioFront), anti-DENV envelope (BioFront), anti-PABPC1 (Proteintech), anti-FLAG (CST), anti-ACTIN (Proteintech), anti-JAK1 (CST), anti-phospho-JAK1 (CST), anti-STAT1 (CST), anti-phospho-STAT1 (CST), anti-PKR (Proteintech), and anti-MX1 (Proteintech) antibodies, and then with secondary antibodies (HRP-conjugated anti-rabbit IgG or HRP-conjugated anti-mouse IgG [CST]). Immunoreactive bands were visualized using a ChemiDoc MP Imaging System (Bio-Rad).

### Isolation of cytoplasmic and nuclear RNAs

The cells were grown in six-well plates, then harvested and resuspended in RLN buffer for cell membrane lysis. After centrifugation at 8,000 rpm at 4°C, supernatants and pellets were collected for cytoplasmic and nuclear RNA extraction, respectively. Cytoplasmic RNA extraction was performed using an RNeasy Mini Kit (Qiagen). Nuclear RNA was extracted using TRIzol reagent (Invitrogen). qRT-PCR was performed to measure the levels of β-actin, U6, and PKR-IT1.

### Immunofluorescence assay

A549 cells (8 × 10^4^) were seeded onto coverslips in 24-well plates and infected with mock or ZIKV at an MOI of 3. At 24 h post-infection, the cells were washed with PBS and fixed in 4% (vol/vol) paraformaldehyde for 30 min. Following permeabilization with 0.02% Triton X-100 and blocking in blocking buffer (5% BSA) for 1 h, the cells were incubated with anti-ZIKV E protein antibody at 4°C overnight. The next day, the cells were incubated with Alexa Fluor 488 conjugated-goat anti-rabbit IgG (H + L) Cross-Adsorbed Secondary Antibody (Thermo) for 1 h, stained with SlowFade Diamond Antifade Mountant with DAPI (Invitrogen), and visualized using an Olympus fluorescence microscope.

### RNA pull-down assay

LncRNA PKR-IT1 and the corresponding vector control were synthesized using an mMESSAGE mMACHINE T7 kit (Invitrogen) according to the manufacturer’s protocol. The RNA pull-down assay was performed using the Pierce RNA-Protein Pull-Down Kit (20614, Thermo Fisher) following the manufacturer’s instructions. A549 cells were transfected with 50 nM biotinylated PKR-IT1 RNA or a control RNA using Lipofectamine 2000. After 24 h, the cells were infected with ZIKV at an MOI of 3. At 24 h post-infection, the cells were collected and lysed in lysis buffer. Biotinylated RNAs labeled at the 3′ end were immobilized on beads and incubated with protein lysates at 4°C overnight. After washing three times and eluting, the pull-down products were resolved by 10% SDS-PAGE, followed by silver staining using the Pierce Silver Stain Kit (Thermo) and Western blotting. Individual gel lanes were excised and subjected to mass spectrometric analysis (Hangzhou Lianchuan Biotechnology Co., Ltd).

### RNA immunoprecipitation (RIP) assay

A549 cells were infected with ZIKV at an MOI of 3. After 24 h, the cells were lysed in RIP lysis buffer (Beyotime), and immunoprecipitation was carried out using an anti-PABPC1 antibody (Proteintech) or equivalent normal IgG (CST) at 4°C overnight. After precipitation, the associated RNA was extracted using TRIzol reagent (Invitrogen). PKR-IT1 and ZIKV RNA levels were assessed by qRT-PCR. The primers used for qRT-PCR are listed in Table S1.

### Statistical analysis

All statistical analyses of host gene levels, viral RNA levels, and viral titers were performed using an unpaired, two-tailed Student’s *t*-test. Data are presented as means ± SD from at least three independent experiments. Pearson correlation analysis was used to assess the strength and direction of associations between different variables. A *P* value of <0.05 was considered statistically significant.

## RESULTS

### ZIKV infection upregulated PKR-IT1 expression in an IFN signaling pathway-dependent manner

To predict the potential functions of PKR-IT1, we carried out a functional annotation of the potential mRNA targets of PKR-IT1 predicted by the LncTarD 2.0 database (38). GO term and KEGG pathway enrichment analyses showed that these mRNAs were mainly involved in the innate immune response, response to virus, and negative regulation of viral genome replication (Fig. S1). Overall, these data suggested that the mRNA targets of PKR-IT1 were closely related to the host response to viral infection.

**Fig 1:**
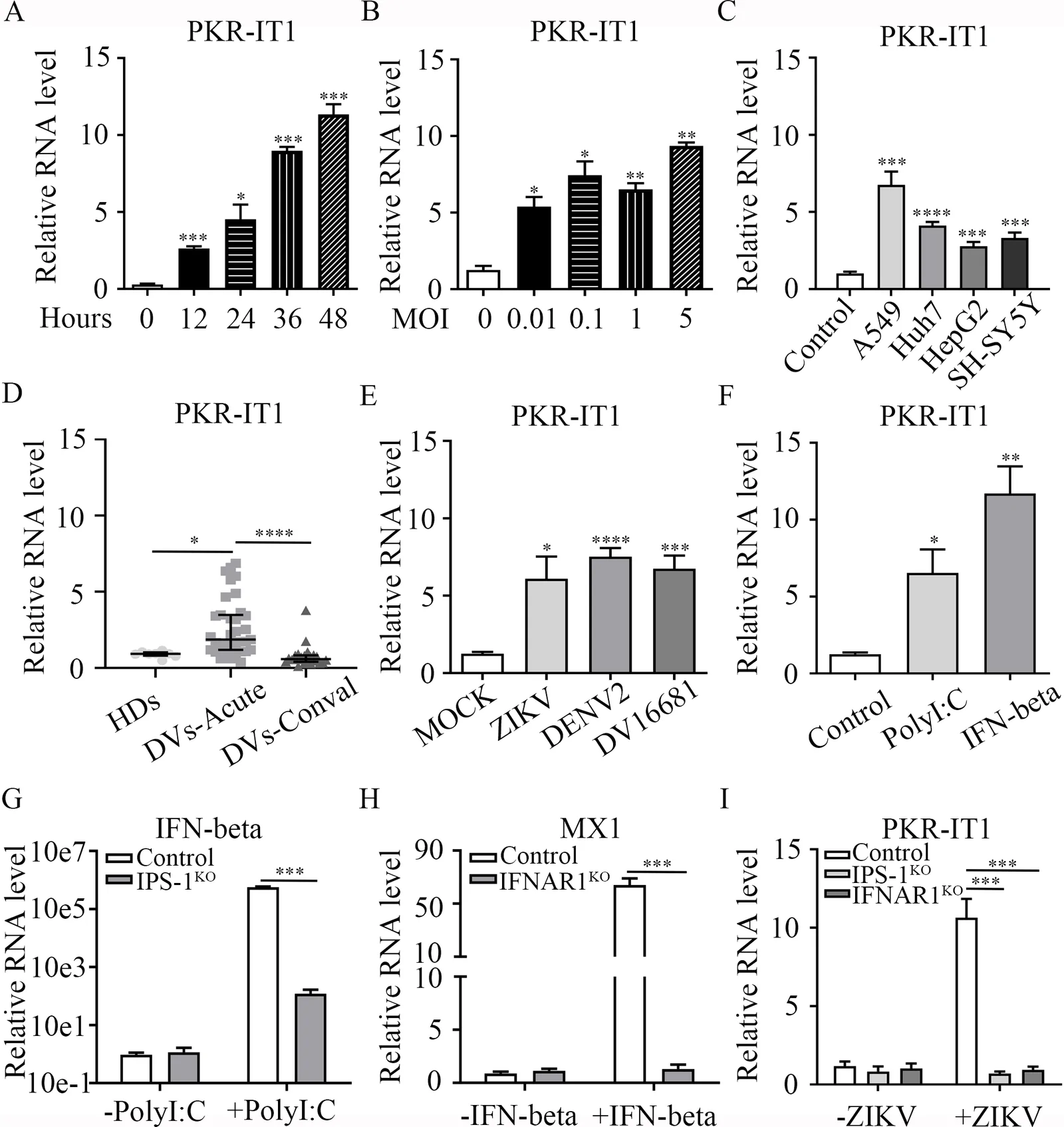
ZIKV-induced expression of PKR-IT1 is regulated by the IFN signaling pathway. (**A**) A549 cells were infected with ZIKV for 0, 12, 24, 36, and 48 h at an MOI 1. (**B**) A549 cells were infected with ZIKV for 24 h at an MOI 0, 0.01, 0.1, 1, and 5. The expression levels of PKR-IT1 were measured by qRT-PCR. (**C**) A549, Huh7, HepG2, and SH-SY5Y cells were infected with ZIKV at an MOI 1. (**D**) The qRT-PCR analysis of PKR-IT1 levels in PBMCs from healthy controls and DENV-infected patients during the acute and convalescent phases. (**E**) A549 cells were infected with ZIKV, DENV2, or DV16681 at an MOI 1. Mock- and virus-infected cells were collected at 24 h post-infection. Total RNAs were extracted for qRT-PCR to detect the levels of PKR-IT1. (**F**) A549 cells were transfected with poly(I:C) for 12 h or treated with 100 units of IFN-β for 20 h. The qRT-PCR was used to measure the levels of PKR-IT1. (**G–I**) Control, IPS-1^KO^, and IFNAR1^KO^ cells were infected with mock, poly(I:C), IFN-β, or ZIKV. Cells were harvested for RNA extraction, and the expression of PKR-IT1 was quantified by qRT-PCR. Actin level was set as an internal control. The data were presented as mean ± SD of at least three independent experiments. *P < 0.05; **P < 0.01; ***P < 0.001; and ****P < 0.0001, unpaired, two-tailed Student’s *t*-test.

In the kinetic assay, the expression of PKR-IT1 progressively increased with increasing infection duration, peaking at 48 h post-infection (Fig. 1A). In addition, PKR-IT1 levels were also correlated with the doses of ZIKV (Fig. 1B), indicating that the expression of PKR-IT1 was triggered by ZIKV in a time- and dose-dependent manner.

As the expression of lncRNAs is generally cell type-specific, we tested whether the expression of PKR-IT1 could be induced in three ZIKV-permissive human cell lines, Huh7, HepG2, and SH-SY5Y. As shown in Fig. 1C, the expression of PKR-IT1 in Huh7, HepG2, and SH-SY5Y cells was significantly upregulated following ZIKV infection. These data suggested that the induction of PKR-IT1 expression by ZIKV is not restricted to a single cell type. In peripheral blood mononuclear cells (PBMCs) from DENV-infected patients, the expression of PKR-IT1 was significantly elevated during the acute phase and decreased to levels comparable to those of healthy individuals during the convalescence phase (Fig. 1D).

Next, we examined whether PKR-IT1 expression was responsive to other viruses using two other flaviviruses (DENV2 and DENV16681). A549 cells were infected with mock or virus (DENV2 and DENV16681) for 24 h, following which total RNA was extracted for qRT-PCR analysis. The results showed that the expression of PKR-IT1 was significantly induced by both DENV2 and DENV16681 (Fig. 1E). These observations indicated that PKR-IT1 is responsive to infection by different flaviviruses.

Given that we found that single-stranded RNA viruses induce the expression of PKR-IT1, we further explored whether its expression is also regulated by dsRNA (39). A549 cells were transfected with the dsRNA analog poly(I:C) and were harvested at 12 h post-transfection for qRT-PCR analysis. The results showed that the expression of PKR-IT1 was upregulated following poly(I:C) stimulation. Considering that the expression levels of type I IFNs were markedly induced by ZIKV in A549 cells, we further investigated whether type I IFNs are involved in the ZIKV-mediated induction of PKR-IT1. The qRT-PCR data demonstrated that IFN-β treatment also enhanced the levels of PKR-IT1, with a greater magnitude of induction than that observed with poly(I:C) or ZIKV (Fig. 1F). This finding implied that the induction of PKR-IT1 may be IFN-dependent. To investigate whether the induction of PKR-IT1 relies on the innate immune response, we employed cell lines deficient in either IPS-1 or IFNAR1 (Fig. 1G and H), two key molecules involved in IFN-mediated innate immunity (40). The levels of PKR-IT1 in control, IPS-1^KO^, and IFNAR1^KO^ cells were compared by qRT-PCR. Under mock conditions, the levels of PKR-IT1 were comparable across all cell types (Fig. 1I). In contrast, ZIKV-induced PKR-IT1 expression was abolished in both IPS-1^KO^ and IFNAR1^KO^ cells. These findings indicated that the expression of PKR-IT1 is differentially regulated by innate immune signaling and is likely an ISG.

### The replication levels of three flaviviruses were decreased in PKR-IT1^OE^ cells

**Fig 2:**
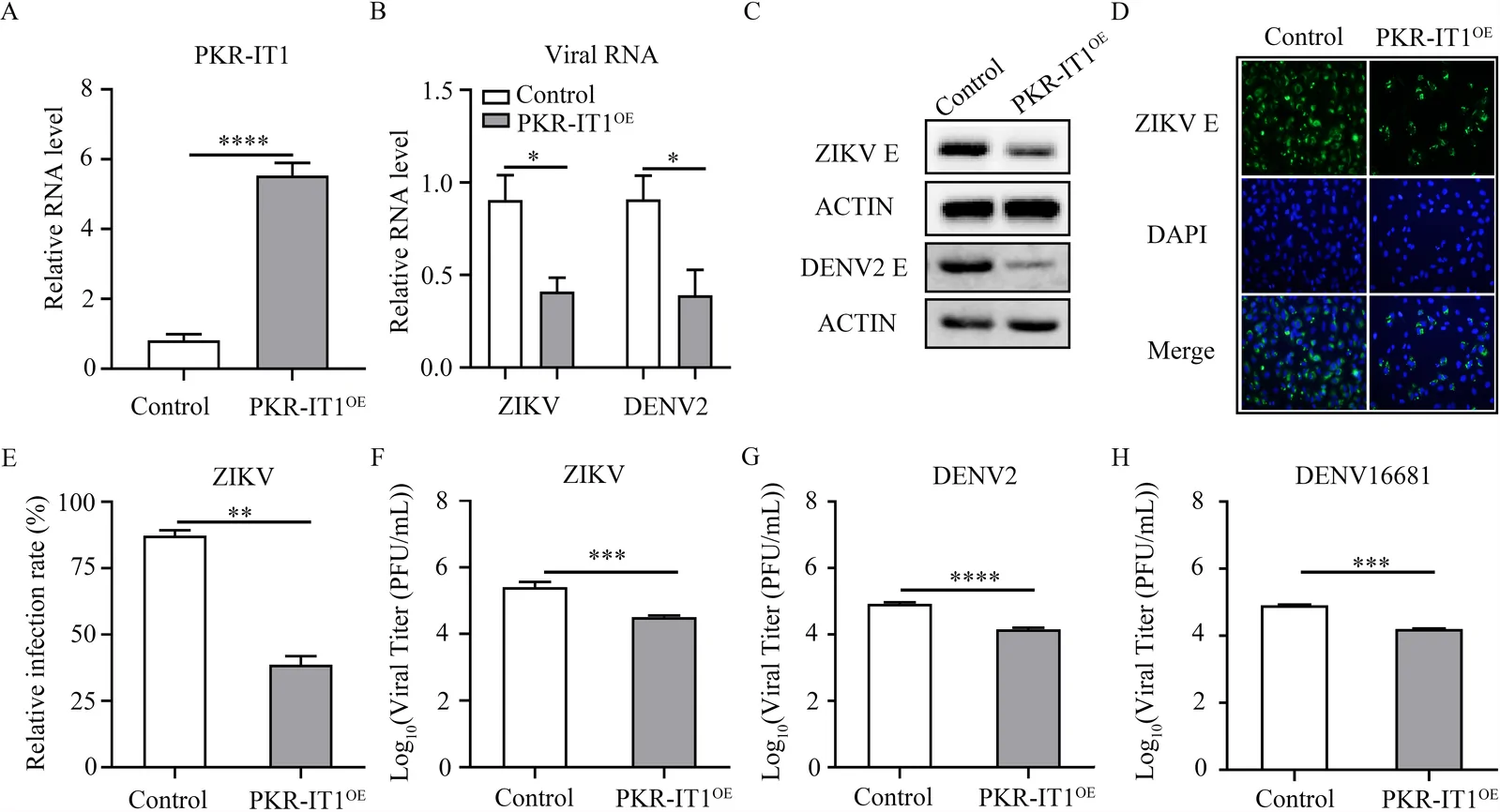
Overexpression of PKR-IT1 inhibits the replication of three flaviviruses. (**A**) The qRT-PCR validation of PKR-IT1 expression in PKR-IT1-overexpressing A549 cells. (**B–H**) Replication levels of flaviviruses. Control and PKR-IT1-expressing cells were infected with ZIKV, DENV2, or DV16681 at an MOI 1. Cells or supernatants were harvested at 24 h post-infection. The viral RNA levels were tested by qRT-PCR (**B**). The viral E protein levels were tested by western blot (**C**) or immunofluorescence microscopy (**D and E**). The viral titers of ZIKV (**F**), DENV2 (**G**), or DV16681 (**H**) were determined by plaque assay. Actin level was set as an internal control. Data were shown as mean ± SD of at least three independent experiments. *P < 0.05; **P < 0.01; ***P < 0.001; and ****P < 0.0001, unpaired, two-tailed Student’s *t*-test. PKR-IT1^OE^, PKR-IT1 overexpressed.

To explore the biologic significance of PKR-IT1, we first examined its role in ZIKV replication using a gain-of-function strategy. A 411 bp PKR-IT1 gene fragment was amplified and inserted into the pcDNA3.1(+) expression vector. Following plasmid transfection, stably transfected A549 cells expressing PKR-IT1 were obtained by G418 selection. The levels of PKR-IT1 in these cells were confirmed by qRT-PCR, which showed that PKR-IT1 expression was upregulated by 6.78-fold relative to control levels (Fig. 2A).

To assess the coding potential of PKR-IT1, we used online prediction tools, including PRIDE Reprocessing 2.0, Translation Initiation Sites, PhyloCSF Score, CPAT Coding Probability, and Bazzini Small ORFs (41). PhyloCSF analysis yielded a negative score for PKR-IT1 regarding its protein-coding ability (Table S2). Similarly, CPAT analysis predicted that PKR-IT1 possesses an extremely low coding potential (Table S2). Furthermore, PRIDE Reprocessing 2.0, Translation Initiation Sites, and Bazzini Small ORFs analyses consistently showed that PKR-IT1 lacks protein-coding potential. To experimentally validate these predictions, we constructed a eukaryotic expression vector expressing full-length PKR-IT1 fused to a FLAG tag (pSG5-PKR-IT1-FLAG). Western blot failed to produce a band in cells transfected with pSG5-PKR-IT1-FLAG, whereas a specific band was detected in pSG5-GFP-FLAG-transfected control cells (Fig. S2A). These data confirmed that PKR-IT1 lacks protein-coding ability.

Next, we evaluated viral replication levels in PKR-IT1-expressing cells by qRT-PCR, western blot, immunofluorescence, and plaque assays. Viral RNA and E protein levels in PKR-IT1-expressing cells were significantly lower than those in control cells (Fig. 2B through E). Consistent with these observations, the viral yields of ZIKV, DENV2, and DENV16681 in PKR-IT1-expressing cells were reduced by 7.98-, 5.89-, and 5.07-fold, respectively (Fig. 2F through H), relative to controls, suggesting that PKR-IT1 exerts antiviral effects against infection by these three flaviviruses, especially ZIKV.

### The silencing or depletion of PKR-IT1 leads to increased viral replication

Next, we performed a cell fractionation assay to examine the subcellular localization of PKR-IT1. As controls, U6 and actin were predominantly localized to the nucleus and cytoplasm, respectively (Fig. S2B and S2C). PKR-IT1 was present in both nuclear and cytoplasmic fractions, and its levels in both compartments gradually increased following viral infection (Fig. S2D).

**Fig 3:**
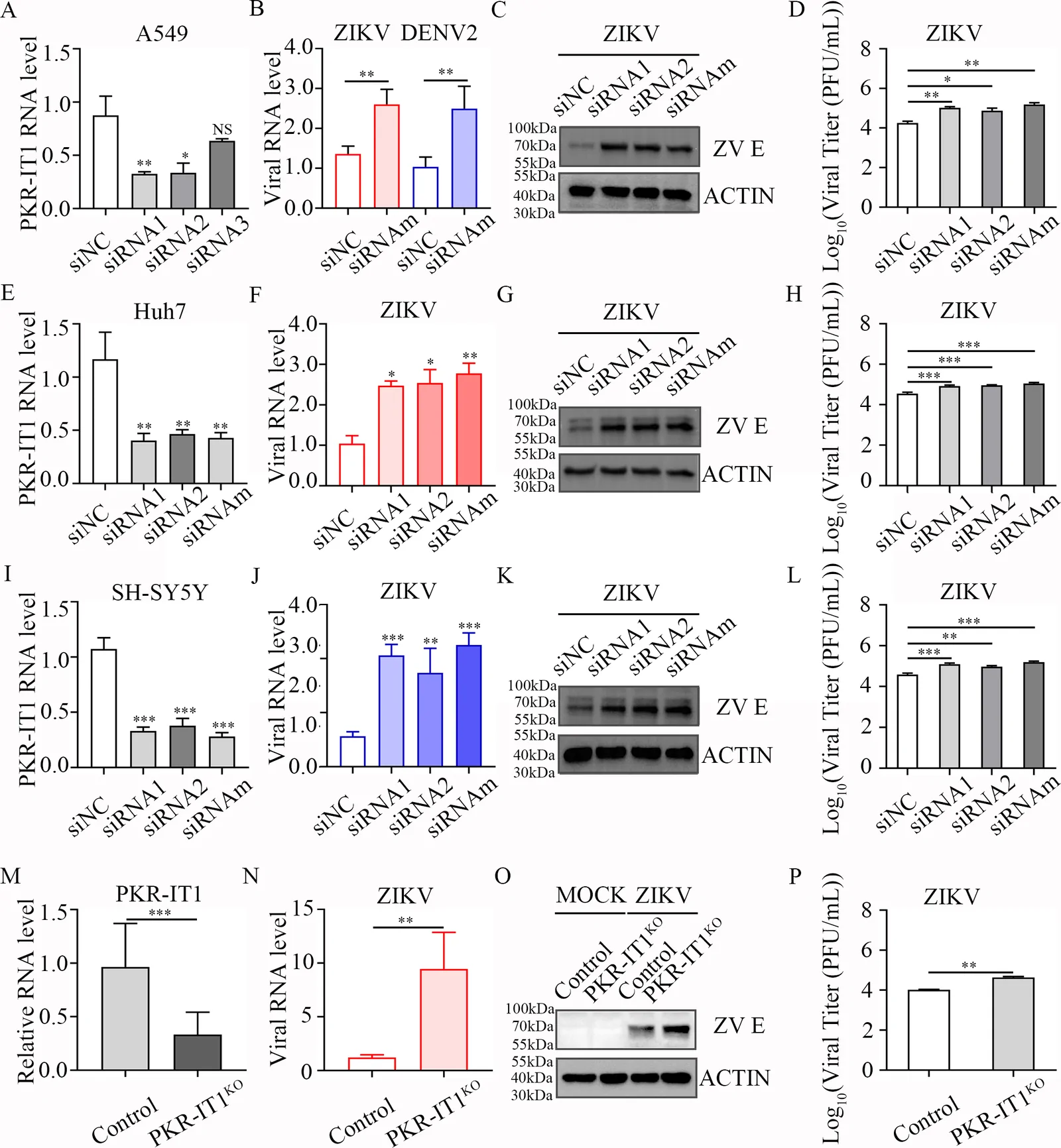
Disruption of PKR-IT1 expression increases the ZIKV replication. (**A–L**) Impact of PKR-IT1 knockdown on the viral replication in A549 (**A–D**), Huh7 (**E–H**) or SH-SY5Y cells (**I–L**). Control and PKR-IT1-silenced cells were infected with ZIKV at an MOI 1. (**M–P**) Impact of PKR-IT1 knockout on the viral replication in A549 cells. Control and PKR-IT1^KO^ cells were infected with ZIKV at an MOI 1. At 24 h post-infection, total RNAs were prepared for qRT-PCR to measure the levels of PKR-IT1 (**A, E, I, and M**) and ZIKV (**B, F, J, and N**). Actin was set as an internal control. The viral E protein levels were tested by western blot (**C, G, K, and O**). Supernatants were harvested at 24 h post-infection for plaque assay (**D, H, L, and P**). Data were shown as mean ± SD of at least three independent experiments. *P < 0.05; **P < 0.01; and ***P < 0.001, unpaired, two-tailed Student's *t*-test. siRNAm, siRNA1+siRNA2.

To further confirm the role of PKR-IT1 in ZIKV replication, we employed two loss-of-function strategies: RNAi-based knockdown and CRISPR/Cas9-mediated gene knockout. In the RNAi-based assay, A549 cells were transfected with different siRNAs. In cells transfected with PKR-IT1 siRNA1 or siRNA2, PKR-IT1 levels were largely silenced (Fig. 3A), while viral RNA concentrations, E protein levels, and viral titers were significantly increased (Fig. 3B through D), demonstrating that PKR-IT1 is a restriction factor for ZIKV. Similarly, in Huh7 and SH-SY5Y cells treated with the two PKR-IT1 siRNAs, PKR-IT1 levels were largely silenced (Fig. 3E and I), whereas viral RNA and E protein levels were significantly increased, as were viral titers (Fig. 3F through H and J through L).

Furthermore, lentivirus carrying two different sgRNAs targeting PKR-IT1 (Fig. S3A) was transduced into A549 cells, followed by selection with puromycin. Genomic DNA was extracted from individual cell clones and prepared for PCR amplification and DNA sequencing to confirm gene disruption. To determine whether the editing of PKR-IT1 affected PKR expression, PKR RNA and protein levels were measured, and were found to be comparable between control and PKR-IT1^KO^ cells (Fig. S3B and C). As expected, the level of PKR-IT1 in PKR-IT1^KO^ cells was only minimally induced by ZIKV infection (Fig. 3M). Subsequently, viral RNA and E protein levels, along with viral titers, were compared between control and PKR-IT1^KO^ cells via qRT-PCR, western blot, and plaque assay, respectively. As shown in Fig. 3N through P, viral RNA, E protein, and viral titers were all significantly higher in PKR-IT1^KO^ cells than in control cells (all *P* < 0.05).

### PKR-IT1 regulates the ZIKV-induced activation of the JAK-STAT pathway

**Fig 4:**
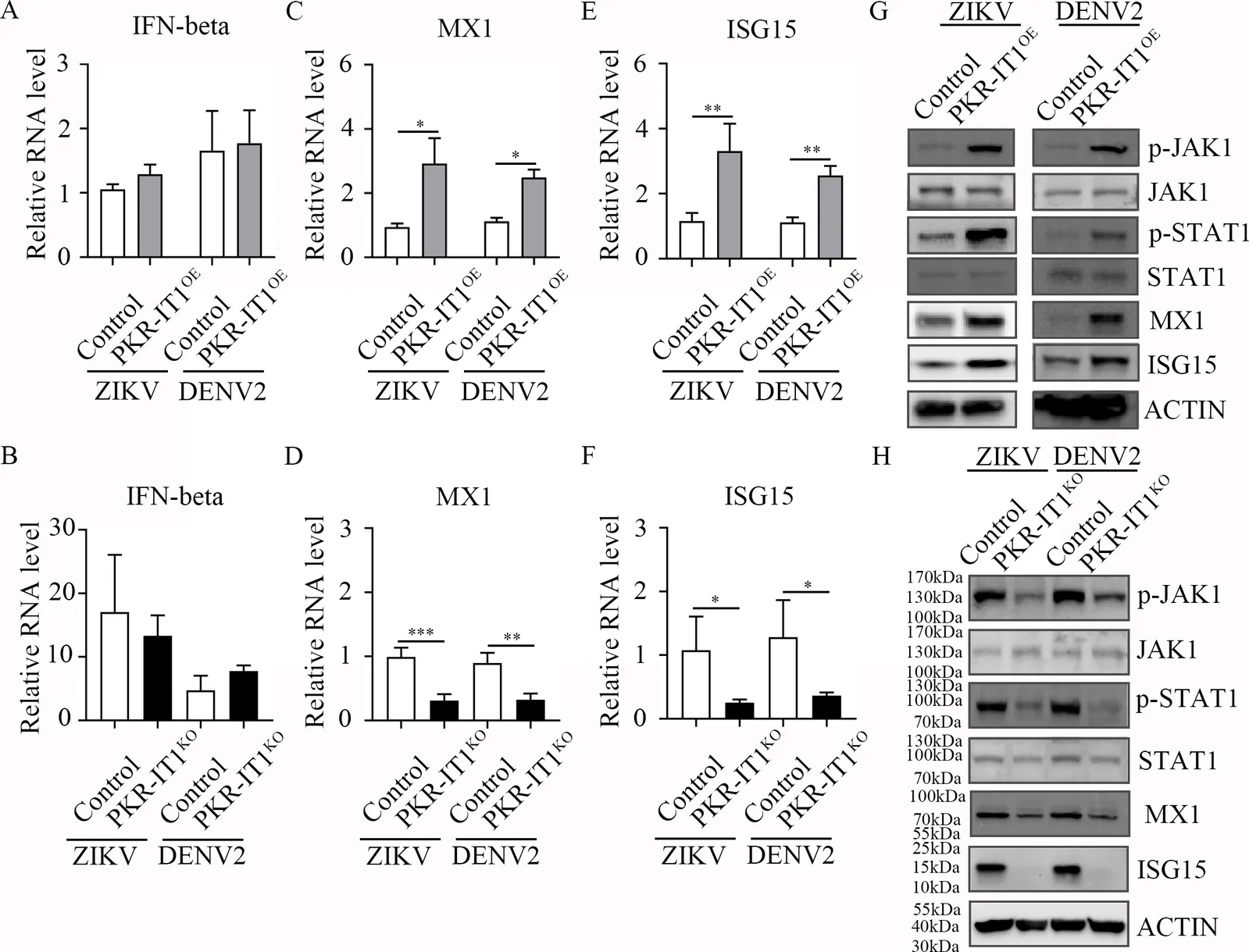
PKR-IT1 positively regulates antiviral immune responses. (**A–F**) The qRT-PCR to detect the levels of IFN-β (**A and B**), MX1 (**C and D**), and ISG15 (**E and F**). The control, PKR-IT1^OE^, and PKR-IT1^KO^ cells were infected with ZIKV or DENV2 at an MOI 1. At 24 h post-infection, total RNAs were prepared for qRT-PCR. Actin level was set as an internal control. (**G and H**) Activation levels of JAK-STAT signaling pathways were tested. Control, PKR-IT1^OE^, and PKR-IT1^KO^ cells were infected with ZIKV or DENV2 at an MOI 1. At 24 h post-infection, the whole-cell extracts were separated by western blot using indicated antibodies. ACTIN was loaded as an internal control. Data were shown as mean ± SD of at least three independent experiments. *P < 0.05, **P < 0.01, and ***P < 0.001, unpaired, two-tailed Student's *t*-test. PKR-IT1^KO^, PKR-IT1 knockout; PKR-IT1^OE^, PKR-IT1 overexpressed.

Next, we investigated whether PKR-IT1 restricts ZIKV infection by regulating the innate immune response, similar to other ISGs or lncRNAs (11, 18, 42). The levels of IFN-β in control, PKR-IT1^OE^, and PKR-IT1^KO^ cells following ZIKV and DENV2 infection were measured by qRT-PCR. No significant difference in the levels of IFN-β was observed between control and PKR-IT1^OE^ cells (Fig. 4A) nor between control and PKR-IT1^KO^ cells (Fig. 4B). Intriguingly, the RNA levels of the classic ISGs, MX1 and ISG15, were significantly elevated in PKR-IT1^OE^ cells compared with controls (Fig. 4C and E), and conversely, they were significantly reduced in PKR-IT1^KO^ cells (Fig. 4D and F).

To determine which transcription factors in the IFN signaling pathway are modulated by PKR-IT1, we examined the activation status of two major transcription factors, JAK1 and STAT1, which undergo phosphorylation upon activation (43). As expected, western blot data revealed that the total protein levels of JAK1 and STAT1 were comparable across all samples (Fig. 4G and H). Following ZIKV or DENV2 infection, the phosphorylation levels of JAK1 and STAT1 and the protein levels of MX1 and ISG15 were noted to be significantly higher in PKR-IT1^OE^ cells than in control cells (Fig. 4G). Furthermore, the phosphorylation levels of JAK1 and STAT1 and the protein expression of MX1 and ISG15 were significantly decreased in PKR-IT1^KO^ cells compared with those in control cells (Fig. 4H). These data indicated that PKR-IT1 positively regulates the activation of JAK1 and STAT1, ultimately promoting ISG induction.

**Fig 5:**
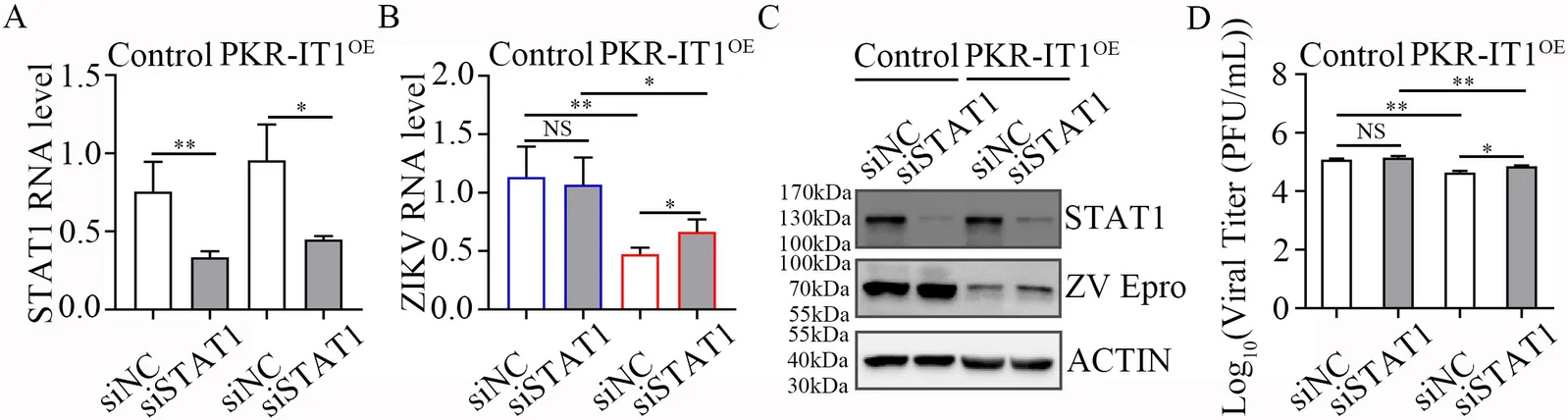
The antiviral activity of PKR-IT1 is partially dependent on its ability to enhance ISG induction. (**A–D**) Control and PKR-IT1^OE^ A549 cells were transfected with siNC or siSTAT1 for 48 h, followed by ZIKV infection (MOI = 1). Cells and supernatants were collected at 24 h. Total RNAs were prepared for qRT-PCR to measure the levels of STAT1 and ZIKV (**A and B**). Actin was set as an internal control. The viral E protein levels were tested by western blot (**C**). The viral titers were measured by plaque assay (**D**). Data are shown as means ± SD from three independent experiments. NS, no statistical significance. *P < 0.05 and **P < 0.01, unpaired, two-tailed Student's *T* test. PKR-IT1^OE^, PKR-IT1 overexpressed.

To confirm whether PKR-IT1 suppresses viral replication through the activation of the JAK-STAT signaling pathway, we employed an RNAi-based approach to silence STAT1 expression. Control and PKR-IT1^OE^ cells were transfected with either siNC or siSTAT1, followed by ZIKV infection. At 24 h post-infection, cellular RNA, proteins, and supernatants were collected for RNA quantification, protein analysis, and viral titration, respectively. In STAT1 knockdown cells, STAT1 expression was largely silenced (Fig. 5A and C). In PKR-IT1^OE^ cells, STAT1 knockdown led to an increase in viral replication, as reflected by viral RNA levels, protein expression, and viral titers; however, these levels remained lower than those observed in control cells (Fig. 5B through D). These results suggested that the antiviral activity of PKR-IT1 is partially dependent on the JAK-STAT pathway activation.

### PKR-IT1 binds to PABPC1 and inhibits its expression

**Fig 6:**
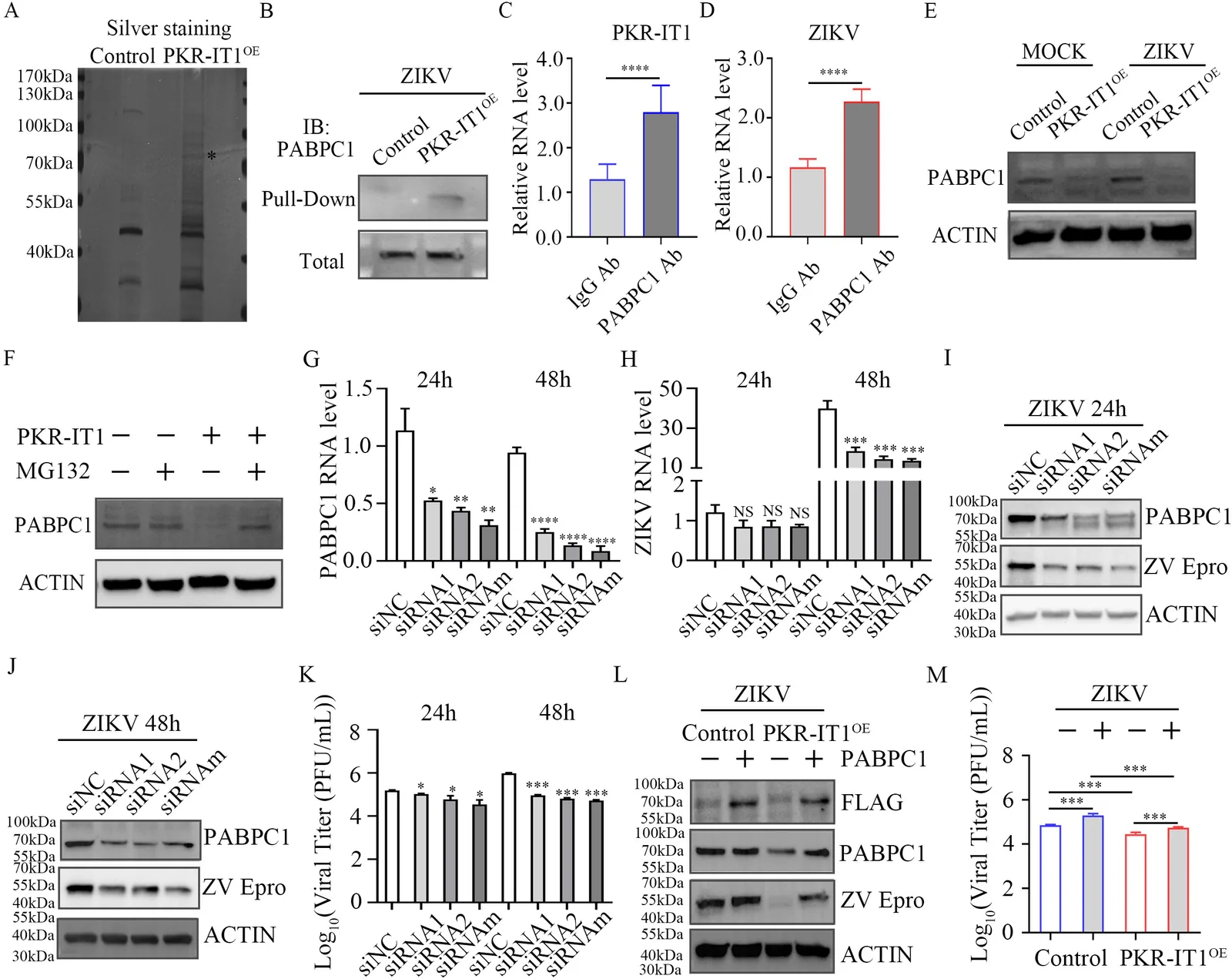
PKR-IT1 can bind to PABPC1 and inhibit its expression. (**A**) Silver-stained gel of proteins pulled down with PKR-IT1 RNA vs vector control in A549 cells by RNA pull-down assay. (**B**) Western blot analysis showed the binding of PKR-IT1 to PABPC1 from RNA pull-down assay. (**C and D**) qRT-PCR from RIP analysis showed PKR-IT1 (**C**) and ZIKV (**D**) in PABPC1 immunoprecipitates and IgG (control) from A549 lysates. (**E**) The PABPC1 protein levels were tested by western blot. (**F**) A549 cells were transfected with vector or pcDNA3.1(+)-PKR-IT1, treated with MG132 for 36 h, and then subjected to western blot to determine PABPC1 protein abundance. (**G–K**) Impact of PABPC1 knockdown on the viral replication in A549 cells. Control and PABPC1-silenced cells were infected with ZIKV at an MOI 1. At 24 and 48 h post-infection, total RNAs were prepared for qRT-PCR to measure the levels of PABPC1 (**G**) and ZIKV (**H**). Actin was set as an internal control. The viral E protein levels were tested at 24 h (**I**) and 48 h post-infection (**J**) by western blot. Supernatants were harvested at 24 and 48 h post-infection for plaque assay (**K**). (**L and M**) Control and PKR-IT1^OE^ cells transiently transfected with vector or PABPC1 plasmid for 24 h, followed by ZIKV infection (MOI = 1). Cells and supernatants were collected at 24 h. The PABPC1 and viral E protein levels were tested by western blot (**L**). The viral titers were measured by plaque assay (**M**). Data are shown as means ± SD from three independent experiments. NS, no statistical significance. *P < 0.05; **P < 0.01; ***P < 0.001; and ****P < 0.0001, unpaired, two-tailed Student’s *t*-test. PKR-IT1^OE^, PKR-IT1 overexpressed; siRNAm, siRNA1 + siRNA2.

Many studies have reported that lncRNAs can modulate gene expression by interacting with specific proteins (44). To identify proteins potentially associated with PKR-IT1, we performed RNA pull-down assays followed by mass spectrometric (MS) analysis. Proteins were initially resolved by SDS-PAGE, visualized via silver staining (Fig. 6A), and subjected to MS. This approach led to the identification of 95 potential PKR-IT1-binding proteins (Table S2), including PABPC1, a PABP family member known to interact with viral RNA and promote the translation of DENV and JEV mRNA (45, 46). Further validation indicated that PABPC1 was readily detected in the PKR-IT1 pull-down complex but not in control samples (Fig. 6B). To further confirm this interaction, RIP assays were performed using antibodies against PABPC1, with non-specific IgG serving as a control. The results showed that PABPC1 binds directly to both PKR-IT1 and ZIKV RNA in A549 cells (Fig. 6C and D). Given the interaction between PKR-IT1 and PABPC1, we investigated whether PKR-IT1 regulates PABPC1 expression. PABPC1 protein levels were assessed by western blot in control and PKR-IT1^OE^ cells following ZIKV infection. It was observed that transfection with a plasmid overexpressing PKR-IT1 accelerated the degradation of PABPC1 protein in A549 cells (Fig. 6E). However, treatment with the proteasome inhibitor MG132 suppressed this degradation (Fig. 6F), supporting the involvement of a proteasome-dependent pathway. Collectively, these results demonstrated that PKR-IT1 can bind to PABPC1 protein and promote its degradation.

Next, we investigated the role of PABPC1 in the regulation of ZIKV replication. Using an RNAi-based knockdown approach, A549 cells were transfected with different PABPC1-targeting siRNAs. In cells transfected with PABPC1 siRNAs, PABPC1 expression was largely silenced (Fig. 6G). In addition, at 24 h post-infection, PABPC1 knockdown led to a significant decrease in ZIKV protein level (Fig. 6I) and viral titer (Fig. 6K), while the viral RNA level was only slightly reduced (Fig. 6H). However, at 48 h post-infection, PABPC1 knockdown led to a substantial decrease in ZIKV replication, including protein level, viral titer, and viral RNA level (Fig. 6H, J and K), demonstrating that PABPC1 functions as a promoter of ZIKV replication.

To determine whether PKR-IT1 exerts its antiviral effect through PABPC1, we performed a rescue experiment. As shown in Fig. 6L and M, supplementation with exogenous PABPC1 attenuated the inhibitory effect of PKR-IT1 overexpression on viral replication, as indicated by E protein expression levels and viral titers. These results suggested that PKR-IT1 suppresses ZIKV replication, at least in part, by interacting with PABPC1 and downregulating its expression.

### Correlation between PKR-IT1 levels in PBMCs of DENV-infected patients and their clinical characteristics

**Fig 7:**
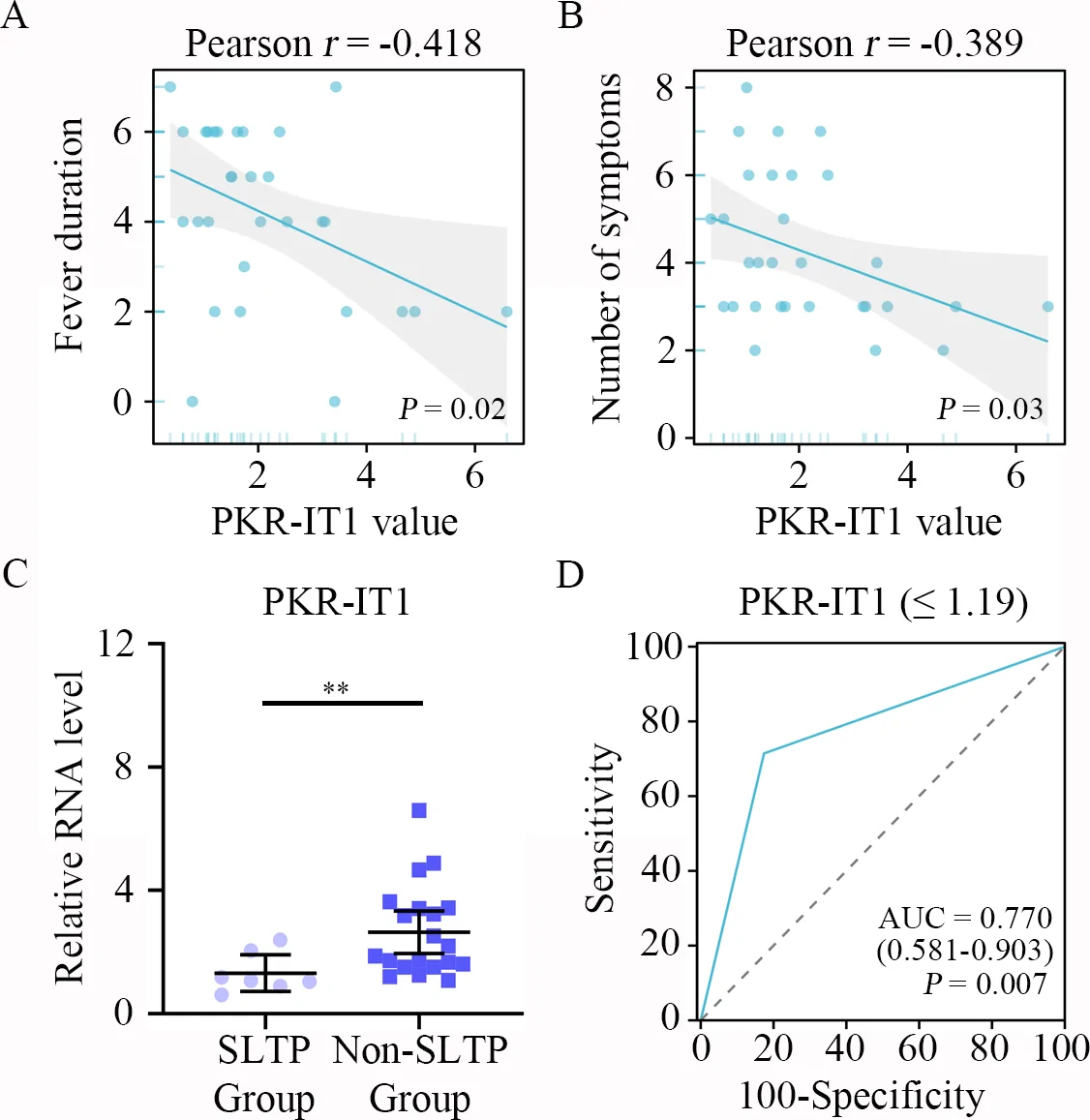
There is a significant negative correlation between PKR-IT1 levels and several clinical features in DENV-infected patients. (**A and B**) Pearson correlation analyses between PKR-IT1 levels and clinical features. (**C**) The expression of PKR-IT1 in PBMCs from DENV-infected patients with severe leukopenia with thrombocytopenia. (**D**) Evaluation of PKR-IT1 expression in predicting the occurrence of severe leukopenia with thrombocytopenia among DENV-infected patients. **P < 0.01, unpaired, two-tailed Student’s *t*-test. SLTP, severe leukopenia with thrombocytopenia.

To better understand the relationship between PKR-IT1 levels and clinical features in DENV-infected patients, we performed a Pearson correlation analysis. The results showed that PKR-IT1 levels were significantly negatively correlated with fever duration (*r* = −0.418, *P* = 0.02) (Fig. 7A) and with the number of symptoms (*r* = −0.389, *P* = 0.03) (Fig. 7B).

Thrombocytopenia (<100 × 10^9^/L) and leukopenia (<4.0 × 10^9^/L) are key indicators in the clinical assessment of dengue severity (47, 48). Our analysis revealed that PKR-IT1 expression was significantly lower in patients with severe leukopenia and thrombocytopenia (SLTP group) than in patients without these manifestations (non-SLTP group) (Fig. 7C).

We next calculated the optimal cut-off value for PKR-IT1 levels to effectively predict the occurrence of SLTP in DENV-infected patients. The optimal threshold was determined to be ≤1.19, corresponding to a sensitivity of 71.43% and a specificity of 82.61% (Table S3). Moreover, receiver operating characteristic analysis was employed to evaluate the ability of PKR-IT1 levels to predict SLTP occurrence. As shown in Fig. 7D, the value of the area under the curve for PKR-IT1 levels was 0.770 (95% CI: 0.581–0.903, *P* < 0.007). In addition, through univariate logistic regression analysis, we identified lower PKR-IT1 expression as a significant predictor of SLTP in DENV-infected patients (OR = 11.88; 95% CI: 1.67–84.52; *P* = 0.013) (Table S4).

## DISCUSSION

Studies have demonstrated that lncRNAs play important roles in the JAK-STAT pathway during viral infection (49, 50). However, the involvement of lncRNAs in the JAK-STAT pathway during flaviviral infection remains largely unknown, as do the putative underlying mechanisms. In the current study, we identified a novel lncRNA, PKR-IT1, which is upregulated upon flavivirus infection via an IFN-dependent mechanism and acts as a regulator of both flavivirus replication and JAK-STAT signaling activation. Furthermore, we clarified the clinical significance of PKR-IT1 levels in DENV-infected patients.

PKR-IT1 is an intronic lncRNA located at the same chromosomal locus as the PKR gene and is distributed in both the cytoplasm and nucleus. A previous report demonstrated that PKR is involved in the antiviral functions of IFN (51). In our study, the expression of PKR-IT1 was regulated in an IFN-dependent manner, suggesting that, like PKR, it is an ISG. Recent studies have identified several ISRs that regulate flavivirus replication through distinct mechanisms. For instance, LncATV promotes viral replication by restricting RIG-I-mediated innate immunity (12); OASL-IT1 restricts ZIKV replication and promotes the activation of three transcription factors in the IFN system upon ZIKV infection (14); SUN2-AS1 enhances flavivirus infection by negatively modulating the expression of essential antiviral ISG proteins (52); and IFN-β-induced ZAP-IT1 inhibits ZIKV replication in an IFN-signaling-independent manner (53). In our study, we found that the antiviral function of PKR-IT1 is partially mediated by the JAK-STAT signaling pathway, distinguishing it from some of the aforementioned ISRs in regulating flavivirus replication.

On the other hand, we found that PKR-IT1, acting as an ISR, upregulates the expression of ISGs (including MX1 (54) and ISG15 (55)) by enhancing JAK-STAT signaling activation during flaviviral infection. This phenomenon is consistent with previous reports showing that ISRs regulate innate antiviral immunity by modulating the expression of distinct subsets of ISGs through diverse mechanisms. For example, lnc-ISG20 functions as a ceRNA that binds miR-326 to relieve its suppression of ISG20 translation (20); OASL-IT1 potentiates p38 MAPK, IRF3, and NF-κB activation to promote ISG induction (14); and LncRNA#32 regulates ISG expression through interaction with ATF2 (21). Taken together, our findings uncover previously unrecognized ISRs that not only expand the functional repertoire of lncRNAs but also provide new insights into the complex regulatory circuitry of innate antiviral immunity.

The functional versatility of lncRNAs is primarily exerted through their interactions with RNA-binding proteins (RBPs), forming dynamic ribonucleoprotein complexes that mediate their biological functions (56). Previous studies have shown that the RNA-binding protein PABPC1 participates in the regulation of flaviviral replication (45, 46). Specifically, PABPC1 binding to the nonpolyadenylated 3’ untranslated region of DENV RNA enhances translation (45). In our study, we also found PABPC1 can bind to ZIKV RNA, while inhibition of PABPC1 attenuated the levels of ZIKV replication. In addition, similar to previous studies (37), treatment with the proteasome inhibitor MG132 suppressed this degradation. This observation suggests that PKR-IT1, in a proteasome-dependent manner, diminishes the role of PABPC1 in ZIKV replication.

In our study, we found that PKR-IT1 was highly expressed in DENV-infected patients compared with healthy donors. Furthermore, a notable negative correlation was observed between PKR-IT1 expression and both fever duration and the number of symptoms. This is potentially attributable to the antiviral properties of PKR-IT1, which may downregulate viral replication during the early phase of DENV infection, thereby decreasing the duration of fever and the number of symptoms. Our results also demonstrated that DENV-infected patients with PKR-IT1 levels ≤1.19 had a significantly higher risk of developing SLTP than those with levels >1.19 at the time of admission. These findings indicated that a lower baseline PKR-IT1 level is independently associated with the occurrence of SLTP. Research has shown that the presence of leukopenia and thrombocytopenia is frequently indicative of disease progression and is associated with dengue severity (47, 48). A study employing machine learning identified a panel of six lncRNAs showing elevated expression during DENV infection that are associated with disease severity (57). These data imply that PKR-IT1 has the potential to serve as a predictive marker for severe dengue fever. However, the potential direct relationship between PKR-IT1 levels and dengue severity remains to be confirmed in larger cohorts.

Our study has several limitations. First, the relationship between PKR-IT1 and the severity of symptoms in DENV-infected patients remains to be established due to a lack of severe cases in our cohort. Second, we could not demonstrate the antiviral role of PKR-IT1 in a mouse model due to poor cross-species conservation of lncRNAs. Further studies are required to verify the function of PKR-IT1 using a wider range of RNA viruses and to determine whether it possesses a broad-spectrum antiviral activity in the host.

### Conclusion

In conclusion, this study is the first to describe the role of PKR-IT1 in flaviviral infection. Flaviviral infection induces PKR-IT1 expression through the IFN signaling pathway. Furthermore, PKR-IT1 attenuates flaviviral replication by interacting with PABPC1, leading to PABPC1 degradation in a proteasome-dependent manner, and promotes the activation of the JAK-STAT signaling cascade. In addition, PKR-IT1 was found to have potential as a diagnostic biomarker for SLTP among DENV-infected patients. We have shown that PKR-IT1 levels first increase and then decrease during viral infections in PBMCs from infected patients compared with those from healthy controls. These findings provide further evidence that host lncRNAs serve as important modulators of both flavivirus replication and the JAK-STAT signaling pathway, and may have utility as diagnostic biomarkers and therapeutic targets for flaviviral infections.

## Data Availability

The mass spectrometry proteomics data have been deposited in the ProteomeXchange Consortium via the iProX partner repository under the data set identifier PXD082814.

## SUPPLEMENTAL MATERIAL

Supplemental figures spectrum.01148-26-s0001.doc Fig. S1 to S3.

Supplemental tables spectrum.01148-26-s0002.doc Tables S1 to S5.

ASM does not own the copyrights to Supplemental Material that may be linked to, or accessed through, an article. The authors have granted ASM a non-exclusive, world-wide license to publish the Supplemental Material files. Please contact the corresponding author directly for reuse.
