# Supplementary material for: The role of interferon-stimulated long non-coding RNA PKR-IT1 in the regulation of Zika virus and dengue virus replication and antiviral response
Source: Microbiol Spectr. 2026 Sep 24;14(11):e01148-26. doi: 10.1128/spectrum.01148-26 (PMC13729175; doi:10.1128/spectrum.01148-26)
Supplement: Supplementary file 1 [file spectrum.01148-26-s0001.doc]

**SFigure legends**

**Figure S1.** GO term and KEGG pathway enrichment analyses of PKR-IT1-targeted mRNAs.

**Figure S2.** Characterization of and subcellular distribution of PKR-IT1.

**Figure S3.** Schematic diagram of CRISPR/Cas9 knockout strategy.
