# Supplementary material for: The role of interferon-stimulated long non-coding RNA PKR-IT1 in the regulation of Zika virus and dengue virus replication and antiviral response
Source: Microbiol Spectr. 2026 Sep 24;14(11):e01148-26. doi: 10.1128/spectrum.01148-26 (PMC13729175; doi:10.1128/spectrum.01148-26)
Supplement: Supplementary file 2 [file spectrum.01148-26-s0002.doc]

**Table S1.** Sequences of all primers were used in this study.

| Primers | Sequence (5’-3’) |
| --- | --- |
| PKR-IT1 QP 5F | AGGCAGTCAGACTCCACAGAT |
| PKR-IT1 QP 3R | GCGTAGAGGTCCACTTCCTTT |
| ZIKV NS1 QP 5F | GTCAGAGCAGCAAAGACAA |
| ZIKV NS1 QP 3R | CAGCCTCCTTTCCCTTAACA |
| DENV2 C QP 5F | TCCTAACAATCCCACCAACAGCA |
| DENV2 C QP 3R | AGTTCTGCGTCTCCTGTTCAAGA |
| IFN-beta QP 5F | AAACTCATGAGCAGTCTGCA |
| IFN-beta QP 3R | AGGAGATCTTCAGTTTCGGAGG |
| Mx1 QP 5F | TTCAGCACCTGATGGCCTATC |
| Mx1 QP 3R | TGGATGATCAAAGGGATGTGG |
| PKR QP 5F | GCAACTCTTTAGTGACCAGC |
| PKR QP 3R | CCGTCAATTCTGTGTTTTGC |
| ISG15 QP 5F | GAGAGGCAGCGAACTCATCT |
| ISG15 QP 3R | CTTCAGCTCTGACACCGACA |
| STAT1 QP 5F | CTAGTGGAGTGGAAGCGGAG |
| STAT1 QP 3R | CACCACAAACGAGCTCTGAA |
| Actin QP 5F | GCTCCTCCTGAGCGCAAG |
| Actin QP 3R | CATCTGCTGGAAGGTGGACA |
| U6 QP 5F | CGCTTCGGCAGCACATATAC |
| U6 QP 3R | CGAATTTGCGTGTCATCCTTG |
| PKR-IT1 BamHI 5F | AGCTCGGATCCATGTTGATCGTTAATTTACAC |
| PKR-IT1 XbaI 3R | GGCCCTCTAGATTAGGTCGATCCTCAGGTTTC |
| PKR-IT1 EcoRI 5F | AGGGCGAATTCATGTTGATCGTTAATTTACA |
| PKR-IT1-FLAG BamHI 3R | GATCTGGATCCTTACTTATCGTCGTCATCCTTGTAATCACCACCACCGGTCGATCCTCAGGTTTCTT |
| sgPKR-IT1 oligo1 | CACCGTTATGTAAGGAGCATAACAA |
| sgPKR-IT1 oligo2 | AAACTTGTTATGCTCCTTACATAAC |

**Table S2**. Analysis of the protein-coding capacity of PKR-IT1.

| Metric | Raw Results | Interpretation |
| --- | --- | --- |
| PRIDE Reprocessing 2.0 | 0 | Non-coding |
| Translation Initiation Sites | 0 | Non-coding |
| PhyloCSF Score | -127.5689 | Non-coding |
| CPAT Coding Probability | 0.0211 | Non-coding |
| Bazzini Small ORFs | 0 | Non-coding |

**Table S3**. RNA-binding proteins pulled down by PKR-IT1.

| Numbers | Protein | Score | Coverage | Unique Peptides | MW [kDa] |
| --- | --- | --- | --- | --- | --- |
| 1 | MYH9 | 27.57 | 7.04 | 13 | 226 |
| 2 | TKT | 27.30 | 22.31 | 9 | 68 |
| 3 | ACTB | 64.74 | 44.53 | 6 | 42 |
| 4 | GAPDH | 24.34 | 34.63 | 6 | 36 |
| 5 | HSPD1 | 27.37 | 17.63 | 5 | 61 |
| 6 | HRNR | 27.28 | 6.67 | 5 | 282 |
| 7 | ENO1 | 22.63 | 20.28 | 5 | 47 |
| 8 | PGD | 10.03 | 10.14 | 4 | 53 |
| 9 | H4C1 | 8.46 | 38.83 | 4 | 11 |
| 10 | RNH1 | 5.65 | 10.20 | 4 | 50 |
| 11 | RDX | 5.48 | 4.80 | 4 | 69 |
| 12 | DSP | 4.82 | 1.67 | 4 | 332 |
| 13 | TUBA1B | 15.56 | 12.64 | 3 | 50 |
| 14 | HSP90AB1 | 13.22 | 6.22 | 3 | 83 |
| 15 | HSPA1B | 7.27 | 8.27 | 3 | 70 |
| 16 | PKM | 6.68 | 8.85 | 3 | 58 |
| 17 | DSG1 | 5.35 | 4.67 | 3 | 114 |
| 18 | PABPC1 | 4.06 | 5.19 | 3 | 71 |
| 19 | ALDH1A1 | 3.93 | 5.19 | 3 | 55 |
| 20 | VIM | 3.13 | 6.44 | 3 | 54 |
| 21 | EEF1A1 | 32.13 | 14.72 | 3 | 50 |
| 22 | EIF5A | 13.10 | 30.52 | 2 | 17 |
| 23 | TUBB4B | 6.52 | 8.99 | 2 | 50 |
| 24 | H2BC12 | 6.31 | 19.05 | 2 | 14 |
| 25 | SBSN | 4.63 | 15.25 | 2 | 61 |
| 26 | FLNA | 3.96 | 1.93 | 2 | 281 |
| 27 | NCL | 3.61 | 2.68 | 2 | 77 |
| 28 | HSPA9 | 3.23 | 3.39 | 2 | 74 |
| 29 | RPS27A | 3.10 | 16.03 | 2 | 18 |
| 30 | RPS23 | 2.76 | 4.35 | 2 | 54 |
| 31 | G6PD | 2.73 | 4.08 | 2 | 59 |
| 32 | ACTC1 | 21.68 | 25.73 | 1 | 42 |
| 33 | H3C1 | 19.74 | 23.53 | 1 | 15 |
| 34 | H3C15 | 15.75 | 23.53 | 1 | 15 |
| 35 | PIP | 8.34 | 17.12 | 1 | 17 |
| 36 | LDHA | 5.55 | 6.02 | 1 | 37 |
| 37 | RPS3A | 4.88 | 6.44 | 1 | 30 |
| 38 | SERPINH1 | 4.60 | 3.59 | 1 | 46 |
| 39 | HSPA5 | 4.55 | 2.75 | 1 | 72 |
| 40 | RPS19 | 4.48 | 8.97 | 1 | 16 |
| 41 | LUM | 4.36 | 4.14 | 1 | 38 |
| 42 | SNRPA | 4.11 | 6.38 | 1 | 31 |
| 43 | P4HB | 4.04 | 5.91 | 1 | 57 |
| 44 | CCT7 | 4.01 | 4.05 | 1 | 59 |
| 45 | PLEC | 3.93 | 0.47 | 1 | 531 |
| 46 | NCBP1 | 3.68 | 1.52 | 1 | 92 |
| 47 | ECH1 | 3.66 | 5.18 | 1 | 36 |
| 48 | IDH1 | 3.36 | 3.14 | 1 | 47 |
| 49 | RPL9 | 3.29 | 14.06 | 1 | 22 |
| 50 | EXOSC7 | 3.23 | 8.25 | 1 | 32 |
| 51 | TPI1 | 3.20 | 6.02 | 1 | 27 |
| 52 | PFN1 | 3.15 | 10.00 | 1 | 15 |
| 53 | EIF4A1 | 2.97 | 4.19 | 1 | 46 |
| 54 | PTBP | 2.79 | 5.27 | 1 | 57 |
| 55 | RPS23 | 2.77 | 18.88 | 1 | 16 |
| 56 | PRSS3 | 2.55 | 4.28 | 1 | 33 |
| 57 | JUP | 2.50 | 1.61 | 1 | 82 |
| 58 | TPM3 | 2.45 | 4.56 | 1 | 33 |
| 59 | ABCC1 | 2.43 | 1.57 | 1 | 171 |
| 60 | LGALS1 | 2.42 | 11.85 | 1 | 15 |
| 61 | FLG2 | 2.37 | 2.09 | 1 | 248 |
| 62 | ALDOA | 2.19 | 8.24 | 1 | 39 |
| 63 | VDAC1 | 2.12 | 8.13 | 1 | 31 |
| 64 | DCD | 2.12 | 10.00 | 1 | 11 |
| 65 | VDAC2 | 2.07 | 7.82 | 1 | 32 |
| 66 | HNRNPA1 | 2.07 | 4.30 | 1 | 39 |
| 67 | LMNA | 1.99 | 1.51 | 1 | 74 |
| 68 | MYL12B | 1.84 | 11.63 | 1 | 20 |
| 69 | ILF3 | 1.79 | 1.45 | 1 | 95 |
| 70 | TMED10 | 1.73 | 9.59 | 1 | 25 |
| 71 | CCT6A | 1.61 | 3.20 | 1 | 58 |
| 72 | AHCY | 1.60 | 2.78 | 1 | 48 |
| 73 | EEF2 | 1.58 | 2.80 | 1 | 95 |
| 74 | PGK1 | 1.44 | 4.32 | 1 | 45 |
| 75 | AZGP1 | 1.42 | 3.36 | 1 | 34 |
| 76 | HNRNPH1 | 1.39 | 4.45 | 1 | 49 |
| 77 | PGAM1 | 1.38 | 5.91 | 1 | 29 |
| 78 | KPNB1 | 1.37 | 2.51 | 1 | 97 |
| 79 | XRCC5 | 1.33 | 1.23 | 1 | 83 |
| 80 | U2AF2 | 1.26 | 2.11 | 1 | 53 |
| 81 | ACTN4 | 1.23 | 1.10 | 1 | 105 |
| 82 | CDSN | 1.19 | 3.40 | 1 | 51 |
| 83 | PHGDH | 1.11 | 3.00 | 1 | 57 |
| 84 | ACTR3 | 1.09 | 2.15 | 1 | 47 |
| 85 | DSC1 | 1.09 | 1.12 | 1 | 100 |
| 86 | CSTA | 1.08 | 12.24 | 1 | 11 |
| 87 | FLNB | 1.06 | 0.92 | 1 | 278 |
| 88 | LDHB | 1.05 | 5.99 | 1 | 37 |
| 89 | ACADVL | 1.04 | 1.98 | 1 | 70 |
| 90 | TGM1 | 1.03 | 0.98 | 1 | 90 |
| 91 | HSD17B10 | 1.02 | 7.66 | 1 | 27 |
| 92 | TXNRD1 | 0.98 | 3.08 | 1 | 71 |
| 93 | VCL | 0.94 | 0.97 | 1 | 124 |
| 94 | RASSF4 | 0.93 | 3.74 | 1 | 37 |
| 95 | HSPA8 | 0.92 | 2.17 | 1 | 71 |

**Table S4**. The optimal cut-off values of PKR-IT1 in predicting the occurrence of severe leukopenia with thrombocytopenia among DENV-infected patients.

| Parameters | Cut-off value | Sensitivity (%) | Specificity (%) | P value |
| --- | --- | --- | --- | --- |
| PKR-IT1 | ≤ 1.19 | 71.43 | 82.61 | 0.023 |

**Table S5**. The PKR-IT1 level was associated with the occurrence of severe leukopenia with thrombocytopenia among DENV-infected patients.

| Parameters | *β* value | OR (95%CI) | *P* value |
| --- | --- | --- | --- |
| PKR-IT1 (≤ 1.19) | 2.47 | 11.88 (1.67, 84.52) | 0.013 |

Note: OR: odds ratio.
